# Analysis of RAMP3 gene polymorphism with body composition and bone density in young and elderly women

**DOI:** 10.1016/j.gene.2019.100009

**Published:** 2019-02-14

**Authors:** Jai Prakash, Maria Herlin, Jitender Kumar, Gaurav Garg, Kristina E. Akesson, Peter S. Grabowski, Tim M. Skerry, Gareth O. Richards, Fiona E.A. McGuigan

**Affiliations:** a Lund University, Dept. of Clinical Sciences Malmö, Clinical and Molecular Osteoporosis Research Unit, Sweden; b Dept. of Environmental Research and Monitoring, Swedish Museum of Natural History, Stockholm, Sweden; c Amity Institute of Biotechnology, Amity University Uttar Pradesh, Noida, India; d Genomics Division, LCGC Life sciences LLP, New Delhi, India; e Skåne University Hospital, Dept. of Orthopedics, Malmö, Sweden; f Human Nutrition Unit, Dept. of Oncology and Metabolism, Sheffield University, UK; g Academic Unit of Bone Biology, Dept. of Oncology and Metabolism, Sheffield University, UK

**Keywords:** RAMP3, Receptor Activity Modifying Protein 3, GPCR, G-Protein Coupled Receptor, KO, Knock-out, SNP, Single Nucleotide Polymorphism, BMD, Bone Mineral Density, CLR, Calcitonin Like-Receptor, CGRP, Calcitonin Gene-Related Peptide, AM1R, Adrenomedullin-1 Receptor, AM2R, Adrenomedullin-2 Receptor, AMY3, Amylin Receptor Complex, OPRA, Osteoporosis Prospective Risk Assessment, LS, Lumbar Spine, FN, Femoral Neck, TB, Total Body, FM, Fat Mass, LM, Lean Mass, RAMP3, SNP, BMD, Fracture, Fat

## Abstract

**Background and aim:**

The Receptor Activity Modifying Proteins (RAMPs) are a group of accessory proteins, of which there are three in humans, that interact with a number of G-protein coupled receptors (GPCR) and play various roles in regulation of endocrine signaling. Studies in RAMP3 knockout (KO) mice reveal an age related phenotype with altered metabolic regulation and high bone mass. To translate these findings into a clinically relevant perspective, we investigated the association between *RAMP3* gene variants, body composition and bone phenotypes in two population-based cohorts of Swedish women.

**Methods:**

Five single nucleotide polymorphisms (SNP) in the vicinity of the RAMP3 gene were genotyped in the PEAK-25 cohort (n = 1061; 25 years) and OPRA (n = 1044; 75 years). Bone mineral density (BMD), fat mass and lean mass (total body; regional) were measured by DXA at baseline, 5 and 10 year follow-up.

**Results:**

BMD did not differ with *RAMP3* genotype in either cohort, although fracture risk was increased in the elderly women (OR 2.695 [95% CI 1.514–4.801]). Fat mass tended to be higher with *RAMP3* SNPs; although only in elderly women. In the young women, changes in BMI and fat mass between ages 25–35 differed by genotype (p = 0.001; p < 0.001).

**Conclusion:**

Variation in *RAMP3* may contribute to age-related changes in body composition and risk of fracture.

## 1 Introduction

Osteoporosis is a common disease in our aging society, affecting one in three women during the course of their lifetime (Melton 3rd et al., 1992). It is characterized by reduced bone mineral density (BMD), and quantitative and qualitative changes to bone tissue, the clinical result of which is an increased risk of fractures (Anonymous, 1993). An important determinant of future skeletal health is the attainment of peak bone mass in young adulthood (Bonjour et al., 1994).

The maintenance of skeletal integrity through bone remodeling is regulated by complex interactions between bone cells and endocrine cells via a number of shared pathways (Karsenty and Oury, 2010; Pei and Tontonoz, 2004). Osteoblasts and adipocytes share a common progenitor, the pluripotent mesenchymal stem cell (Hu et al., 2018a), with the balance between osteogenesis and adipogenesis changing with age, resulting in a shift towards adipocytes (Berendsen and Olsen, 2014; Chen et al., 2016). At the population level this is reflected in proportionally higher fat mass, even without a change in body weight, while the distribution of fat mass also changes with age (Prentice and Jebb, 2001; Leal et al., 2015; Chantler et al., 2016).

Given the mechanistic links between fat and bone metabolism, the identification of pleiotropic genes may offer a deeper understanding of the pathogenesis and underlying genetic architecture of osteoporosis (Liu et al., 2009; Medina-Gomez et al., 2017; Hu et al., 2018b). In contrast to the hypothesis-free approach of GWAS, the rationale for the present candidate gene study is based on evidence from a mouse model, for a high bone mass phenotype (Pacharne et al., 2011) and reduced propensity to become obese with age (Dackor et al., 2007). This knockout (KO) lacked the gene for an accessory protein receptor activity modifying protein 3 (RAMP3) involved in signaling by the hormones adrenomedullin and amylin.

Three mammalian RAMPs are known in humans. RAMPs are single-pass transmembrane proteins, and an important family member of G protein-coupled receptor (GPCR) accessory proteins (Hay et al., 2006; Routledge et al., 2017). GPCRs recognize cell surface ligands to initiate intracellular signaling (The state of GPCR Research in 2004, 2004) and RAMPs modulate their pharmacology, trafficking and signaling properties. RAMPs were first shown to interact with the calcitonin like-receptor (CLR), so that CLR + RAMP1 forms a receptor for calcitonin gene-related peptide (CGRP) a peptide involved in pain perception and vasodilator function. RAMPs 2 and 3 associate with the CLR to form two distinct Adrenomedullin receptors. The CLR + RAMP2 Adrenomedullin-1 receptor (AM1R) is a potent vasodilator and has functions in angiogenesis and a range of diseases. The CLR + RAMP3 Adrenomedullin-2 receptor (AM2R) is less well characterized. Interaction of RAMP3 with the calcitonin receptor, produces an amylin receptor complex (AMY3) (Routledge et al., 2017; The state of GPCR Research in 2004, 2004). RAMPs have also been show to interact with a number of other GPCRs, and are predicted to have many more unknown partners (Barbash et al., 2017).

Knockout of the various RAMPs in rodent models has established distinct physiological functions throughout the life course. Targeted deletion of RAMP3 demonstrated that it has an important role in regulating body weight with increased age; the mice lacking RAMP3 appeared normal until reaching old age, after which time their weight decreased (Dackor et al., 2007; Bailey et al., 2010). Furthermore, it has also been shown that aging RAMP3 null mice have accelerated bone development and higher bone mass compared with wild type controls. Also, in response to mechanical loading, RAMP3 knockouts produce a greater adaptive response as measured by more periosteal formation (Livesey et al., 2013). This suggests that RAMP3 acts as a negative modulator of bone adaptation, which can be explained in terms of keeping bone mass to an appropriate level, and avoiding an over-engineered skeleton which would be expensive to grow, maintain and use. At the population level, human *RAMP3* variants have not been studied extensively, although functional characterization of two single nucleotide polymorphisms (SNPs) in the human RAMP3 protein has been performed (Bailey et al., 2010).

The rationale for our study was to comprehensively evaluate the association between selected *RAMP3* SNPs with bone and body composition phenotypes in the setting of a prospective study design. We hypothesized that *RAMP3* variants would be associated with 1) body composition, 2) change in body composition and 3) bone phenotypes including fracture. Moreover, we also explore 4) age related differences in the contribution of *RAMP3* SNPs to these phenotypes in two differently aged cohorts of women; young adults and elderly women.

## 2 Materials and methods

### 2.1 Subjects

Two population based cohorts of Swedish women were studied; the OPRA (Osteoporosis Prospective Risk Assessment) cohort consisting of 1044 elderly women all aged exactly 75 (75.2 ± 0.1) at the time of recruitment and the PEAK-25 cohort consisting of 1061 women all aged exactly 25 years (25.5 + 0.2).

The OPRA cohort was prospectively followed with reassessment at 5 years (n = 715) and 10 years (n = 382); the PEAK-25 cohort returned for assessment after 10 years (n = 731).

Informed consent was obtained from all individual participants included in the study and all procedures performed were in accordance with the ethical standards of the Regional Ethical Review Board in Lund and with the 1964 Helsinki declaration and its later amendments or comparable ethical standards.

### 2.2 Measurement of BMD and body composition using DXA

BMD and body composition were measured using dual-energy x-ray absorptiometry (OPRA: Lunar DPX-L; PEAK-25: Lunar Prodigy (Lunar Corporation, Madison, WI, USA)). BMD (g/cm^2^) was measured at the Lumbar spine (LS), femoral neck (FN) and total body (TB). Lumbar spine from OPRA participants was not included in the analyses in due to the high incidence of degenerative changes (Tenne et al., 2013). Body composition measures included fat mass (FM, kg) and lean mass (LM, kg) measured at total body (TB), trunk and leg.

Calibrations were performed daily using a phantom supplied by the manufacturer. Precision error for bone density at baseline was 0.94% and 1.45% for total body and lumbar spine respectively in the OPRA cohort (Lenora et al., 2010) and 0.90% and 0.65% for femoral neck and lumbar spine respectively in PEAK-25 (Callreus et al., 2012).

Coefficients of variation for body composition were 30.2% and 10.6% for total body fat and total body lean mass respectively in OPRA; and 39.6% and 11.6% respectively in PEAK-25. For the OPRA cohort, all measurements at baseline were performed using the same instrument, while analyses of scans were made with software versions 1.33 and 1.35. For PEAK-25, the same instrument was used throughout, with software versions 2.15–7.70.

Additional phenotypes measured included weight (kg); height (cm) and BMI (kg\m^2^). The ratio between fat mass and lean mass was calculated, for total body and trunk fat.

### 2.3 Fracture

In young women fracture incidence is low; therefore, fracture data is analyzed only in OPRA. Information on fractures was continuously registered through the X-rays files at the Radiology Department, Malmö, Skåne University Hospital, as previously described in detail (Buchebner et al., 2014). This department serves the Department of Orthopedics, the only unit treating adult and pediatric fractures in the catchment area, hence loss to follow-up is low (Jonsson et al., 1994). Prevalent fractures (i.e., prior to inclusion in the study at age 75) were registered, as previously reported (Gerdhem and Akesson, 2007).

In the current analyses however we report only on incident fracture data collected until October 31, 2012, providing a maximum follow-up for fracture of 17.2 years (mean 13.1 years). Fractures resulting from pathology and high energy trauma were excluded. In this report, our primary outcomes were ‘any fracture’, ‘major osteoporotic fracture’ (i.e., hip, vertebra, distal radius, and shoulder) and ‘hip fracture’ as a group and per fracture.

### 2.4 Blood sample collection and genotyping

Non-fasting blood was collected before noon for DNA isolation and stored at -80^0^c until analysis. Total genomic DNA was isolated using the QIAamp 96 DNA blood kit (Qiagen, Valencia, CA, USA).

### 2.5 Genotyping

Five SNPs in and around the *RAMP3* gene (Chromosome 7p13) were genotyped in both cohorts (Table 1). SNPs were selected from ensembl (http://www.ensembl.org).

**Table 1:** RAMP3 SNPs genotyped in this study.

SNP (Major/Minor Alleles)	Position (in chromosomal order)	Description
rs3757575 (G/A)	Chr 7: 45156136	Upstream; intergenic
rs2074654 (T/C)	Chr 7: 45177416	Exon 2; non-synonymous (missense)
rs1294935 (T/C)^a^	Chr 7: 45178767	Intron 2; tag SNP
rs11982639 (C/G)	Chr 7: 45183419	3’UTR; splice site
rs12702121 (A/G)	Chr 7: 45183660	Exon 3; tag SNP

a Minor allele in Ensembl is T.

Genotyping was performed using Taqman SNP genotyping Assay (Applied Biosystems, Foster City, CA, USA). PCR was conducted in a Dual 384-well GeneAmp PCR system 9700, with an endpoint plate read on ABI 7900HT using the SDS 2.2.2 software (Applied Biosystems). Genotyping was performed blind and approximately 3% of samples from each cohort were genotyped in duplicate with 100% concordance. DNA was available for 1004 (OPRA) and 1006 (PEAK-25) individuals. Genotyping was successful in 997 to 1002 women (OPRA) and 999 to 1002 women (PEAK-25); an overall success rate of >98%. Departures from Hardy-Weinberg equilibrium (HWE) were tested for each SNP using the Chi^2^ test with one degree of freedom (HWE Program, Jurg Ott and Rockefeller University, New York). All SNPs followed HWE (p ≥ 0.05).

### 2.6 Statistical analysis

Genotype specific differences between phenotypes were analyzed with the Kruskal-Wallis test using dominant models (comparing the major allele homozygotes Vs. heterozygotes + minor allele homozygotes) and recessive models (comparing the major allele homozygotes + heterozygotes allele Vs. minor allele homozygotes). Regression analysis was performed to determine association between SNPs and phenotypes, adjusting for confounders as appropriate (body size (i.e. Height^2^), TBFM, BMD, smoking). The Chi^2^ test was used to analyze association between genotypes and categorical variables (e.g. fracture); while linear regression was used to identify and adjust for confounding factors. Age was not adjusted for since all participants within each cohort were the same age. A priori power analyses, assuming a SD of 0.13 g/cm^2^ in BMD, indicated that our sample size allowed >80% power to detect differences of 0.065 g/cm^2^ between genotypes assuming a minor allele frequency of >0.21.

The phenotypes and markers studied are not fully independent therefore applying a Bonferroni correction would be over-stringent. We report uncorrected p-values (two-tailed), acknowledging that multiple tests were performed. Nominal significance was considered with p < 0.05. Statistical analysis was performed with SPSS (v20.0, SPSS Inc., Chicago, IL).

## 3 Results

*RAMP3* genotype and minor allele frequencies did not differ between the cohorts (Table 2). The general and clinical characteristics of the participants from the two differently aged cohorts of women are reported in Table 3. As expected, in comparison to the elderly OPRA participants, the young PEAK-25 women had higher lean mass and lower fat mass values. This is reflected by a higher ratio of fat to lean mass -overall and at the trunk, in the older women.

**Table 2:** *RAMP3* genotype frequencies in the PEAK-25 and OPRA cohorts.

SNP & Alleles	PEAK-25				OPRA			
	Homozygotes Major Allele No. (%)	Heterozygotes No. (%)	Homozygotes Minor Allele No. (%)	MAF	Homozygotes Major Allele No. (%)	Heterozygotes No. (%)	Homozygotes Minor Allele No. (%)	MAF
rs3757575 (G/A)	592 (59.3%)	365 (36.5%)	42 (4.2%)	0.23	622 (62.3%)	334 (33.4%)	43 (4.3%)	0.21
rs2074654 (T/C)	905 (90.3%)	93 (9.3%)	4 (0.4%)	0.05	933 (93.2%)	65 (6.5%)	3 (0.3%)	0.04
rs1294935 (T/C)	260 (26.0%)	484 (48.4%)	255 (25.5%)	0.50	234 (23.4%)	496 (49.7%)	268 (26.9%)	0.48
rs11982639 (C/G)	594 (59.4%)	340 (34.0%)	66 (6.6%)	0.24	617 (61.9%)	332 (33.3%)	48 (4.8%)	0.21
rs12702121 (A/G)	595 (59.4%)	341 (34.0%)	66 (6.6%)	0.24	619 (61.8%)	334 (33.3%)	49 (4.9%)	0.22

**Table 3:** Anthropometric and clinical characteristics of the OPRA and PEAK-25 cohorts at baseline.

Variable	N	PEAK-25	N	OPRA
Age (yrs)	1060	25.5 (0.20)	1044	75.23 (0.15)
Weight (kg)	1060	64.7 (11.40)	1044	67.78 (11.68)
Height (cm)	1060	167.6 (6.08)	1024	160.52 (5.71)
BMI (kg/m^2^)	1060	23.04 (3.81)	1024	26.27 (4.12)
Current/former smoker	1060	457 (43.4%)	1044	354 (33.9%)
BMD (g/cm^2^)				
Femoral neck	1058	1.053 (0.123)	947	0.765 (0.138)
Total hip		Not available	924	0.849 (0.149)
Total body	1060	1.174 (0.073)	931	1.006 (0.098)
Lumbar spine	1060	1.239 (0.131)	976	0.884 (0.170)
Body Composition (kg)				
Total Body - Fat mass	1060	21.22 (8.40)	931	26.09 (7.90)
Total Body - Lean mass	1060	40.38 (4.68)	931	37.28 (3.95)
Total Body - Ratio Fat/Lean	1060	0.53 (0.19)	931	0.70 (0.19)
Trunk - Fat mass	1060	10.22 (4.65)	931	12.57 (3.93)
Trunk - Lean mass	1060	19.71 (2.48)	931	19.00 (2.18)
Trunk - Ratio Fat/Lean	1060	0.52 (0.21)	931	0.66 (0.18)

Values are mean (standard deviation).

### 3.1 Association of RAMP3 SNPs with body composition

We observed age related differences between *RAMP3* SNPs and the phenotypes studied. In OPRA, carriers of SNP rs2074654 minor ‘C' allele tended towards slightly higher values of both fat (TB-FM 2.21% difference), and lean mass (TB-LM 1.34%), although after adjustment for body size, p-values increased at some sites (Table 4). The ratio of fat to lean mass did not differ. In the PEAK-25 cohort, after adjustment for body size, body composition did not differ with *RAMP3* genotype (data not shown).

**Table 4:** Association of *RAMP3* rs2074654 with *BODY COMPOSITION* in elderly women (OPRA cohort).

OPRA Variable at Baseline (75 yrs)	rs2074654 ‘TT’ Homozygotes (n = 842)	rs2074654’C′ Allele Carriers (n = 61)	Difference	P-value^a^	P-value^b^ (adjusted)
Fat Mass (kg)					
Total body	25.96 (0.28)	28.17 (0.82)	2.21	0.024	0.065
Trunk	12.50 (0.14)	13.73 (0.41)	1.23	0.018	0.035
Leg	9.06 (0.11)	9.75 (0.31)	0.69	0.035	0.147
Lean mass (kg)					
Total body	37.23 (0.14)	38.57 (0.54)	1.34	0.015	0.041
Trunk	18.98 (0.08)	19.60 (0.28)	0.62	0.029	0.117
Leg	12.03 (0.05)	12.60 (0.21)	0.57	0.011	0.038
Ratio Fat\Lean mass					
Total body	0.70 (0.01)	0.73 (0.02)	0.30	0.129	0.144
Trunk	0.70 (0.01)	0.73 (0.02)	0.03	0.129	0.144
Leg	0.75 (0.01)	0.78 (0.02)	0.03	0.163	0.275

Association analyzed using the dominant model (comparing major allele homozygotes Vs. heterozygotes + minor allele homozygotes). Reported values, mean (SE).

a Kruskal-Wallis.

b Linear regression - adjusted for body size.

### 3.2 Association of RAMP3 SNPs with change in body composition

We investigated whether *RAMP3* SNPs were associated with change in body composition over time; over 5-years for the elderly women and 10-years in the young women. In the elderly women, changes in body composition, between the ages of 75–80, did not differ with genotype for any of the SNPs (Supplementary Table S1).

In contrast, in the young PEAK-25 cohort, changes in BMI and fat mass over 10-years i.e. between ages 25 and 35 differed with genotype. Individuals carrying the minor ‘A' allele of SNP rs3757575 increased with respect to BMI, overall fat mass and trunk adiposity and the ratio of fat to lean mass also increased. Lean mass however did not differ appreciably with genotype (Table 5).

**Table 5:** RAMP3 rs3757575 association with *BODY COMPOSITION CHANGE OVER TIME* in young women (PEAK-25).

PEAK-25 10 year change (between 25y-35y)	rs3757575 ‘G' Allele Carriers (n = 649)	rs3757575 ‘AA' Homozygotes (n = 30)	P-value^a^	P-value^b^ (adjusted)
BMI	0.96 (0.01)	1.08 (0.06)	0.040	0.002
Total Body Fat mass (kg)	−3.30 (0.49)	4.23 (2.78)	0.017	0.001
Total Body Lean mass (kg)	4.02 (0.19)	4.01 (0.77)	0.857	0.833
Change in ratio Fat\Lean mass	1.01 (0.02)	1.47 (0.20)	0.015	<0.001
Trunk Fat mass (kg)	−1.44 (0.29)	3.00 (1.49)	0.014	0.001
Trunk Lean mass (kg)	−1.26 (0.13)	−0.97 (0.48)	0.610	0.444
Change in ratio Fat\Lean mass	1.16 (0.03)	1.74 (0.27)	0.013	<0.001

Association analyzed using the recessive model (comparing major allele homozygotes + Heterozygotes Vs. minor allele homozygotes). Reported values are mean (Std Error).

a Kruskal-Wallis.

b Linear regression - after adjustment for body size.

### 3.3 Association of RAMP3 SNPs with skeletal phenotypes

In the elderly women, risk of any type of fracture was higher among carriers of the minor ‘C' allele of SNP rs2074654 independent of bone density and fat mass (OR_adjusted_ 2.695 [95% CI 1.514–4.801]. Osteoporotic fracture risk was also elevated, although only after adjustment, while hip fracture did not differ (Table 6).

**Table 6:** Association of RAMP3 rs2074654 with baseline FRACTURE in elderly women (OPRA cohort).

Fracture type	rs2074654 Genotype	WITHOUT fracture No. (%)	WITH fracture No. (%)	Odds ratio (95% CI)	P-value^a^	Odds ratio (95% CI)^2^	P-value^2^
Any	TT	474 (95.4)	459 (91.1)				
	TC + CC	23 (4.6)	45 (8.9)	2.02 (1.20–3.39)	0.008	2.620 (1.46–4.69)	0.001
Osteoporotic^1^	TT	515 (94.3)	418 (91.9)				
	TC + CC	31 (5.7)	37 (8.1)	1.47 (0.90–2.41)	0.126	1.846 (1.07–3.18)	0.022
Hip	TT	756 (93.1)	177 (93.7)				
	TC + CC	56 (6.9)	12 (6.3)	0.92 (0.48–1.74)	0.788	0.962 (0.49–1.90)	0.934

1 Osteoporotic fracture sites include hip, vertebrae, distal radius, proximal humerus.

2 Binary logistic regression adjusted for smoking, total body fat mass and total body BMD at baseline.

a Kruskal-Wallis.

In the OPRA cohort, BMD did not differ with rs2074654 genotype at any measured site (FN: 0.763 Vs 0.785, p = 0.22) nor with the other studied SNPs or with change in BMD between the ages of 75–80 (data not shown). In the PEAK-25 cohort, after adjustment for body size, no differences in bone density or with change in BMD between ages 25 and 35 (data not shown) were observed.

## 4 Discussion

The objective of the present study was to determine the association between *RAMP3* variants with body composition, bone density and fracture. The basis for the study lies in the manifestation of a bone and body composition phenotype, which differed with age, in a knockout mouse model (Pacharne et al., 2011) and the assumption that key regulatory genes are shared across species. In this candidate gene association study we specifically wanted to address the temporal aspect and determine if association differed at young and old age and additionally whether *RAMP3* genotype was related to change in these phenotypes over 5 or 10 years of aging.

Results from the study tentatively suggest that *RAMP3* SNPs may play a role, albeit minor, in regulating body composition and influence fracture risk. We found that levels of both fat and lean mass differed with variation in *RAMP3*, although only in the elderly women. Given that the cohorts were similarly sized, we assume that this lack of replication is not a question of study power, but rather reflects an age dependent relationship. This is supported by observations from other studies that the effect size of weight susceptibility genes differs across the life course (Kvaloy et al., 2013). We also observed that in the young women change in fat mass differed with *RAMP3* variation; carriers of the common allele gaining less fat overall and at the trunk (i.e. maintaining a leaner phenotype). In very old age, there was no genotype related difference in change-in-fat-mass over the five years studied. We reason that this possibly is because at older ages environmental and lifestyle factors along with overall health status have a stronger impact than genetic factors in maintaining a stable weight. This is in line with the reduced predictability with advancing age also noted for specific biomarkers. Although the mechanisms underlying the results of this present study are unclear, in an animal study (Dackor et al., 2007) Dackor et al. reported that RAMP3 plays an important role in maintaining normal body weight with age. The results of the present study seem to support this finding. We speculate that RAMPs act to mediate the basal effects of normal GPCR signaling, and that under physiological conditions or disease RAMP3 may become induced to alter the signaling of GPCRs (Kvaloy et al., 2013; Hewitt et al., 2005; Gibbons et al., 2007; Ono et al., 2000).

Contrary to our hypothesis that variation in the *RAMP3* gene could be associated with bone density, because of the interdependence with body weight and fat mass, bone density did not differ between genotypes at any age. Despite this, risk of fracture in the elderly women increased, apparently independently of BMD and fat mass. This may mirror factors not measured with the techniques used, such as qualitative aspects of cortical and trabecular bone. Alternatively, through effects on muscle function by GPCR (White, 2016) which might lead to falls. Association with osteoporosis related phenotypes is feasible. There are a number of functionally-relevant *RAMP3* variants in humans and in our study rs3757575 is an upstream intergenic SNP that may affect transcription while rs2074654 is exonic, non-synonymous and has the potential to alter gene expression. Indeed, this latter SNP, despite its low frequency is predicted to be deleterious, based on disruption of the protein structure or interference with its function or interaction (Bailey et al., 2010; Burke et al., 2007). Typically, in such cases risk of developing the disease can be increased even if not itself causal. It should also be kept in mind that there may be species differences in the roles of RAMP3, both in relation to specific modulation of a single GPCR interacting partner and in the relative changes induced by the complex interactions of RAMP3 with all its partners. Dissection of these complex relationships is out with the scope of this study.

Limitations of this study are acknowledged, the first being that this is a candidate gene approach and the reported associations modest at best. Although GWAS has been very successful in identifying loci, the majority as yet have no proven biological role in bone metabolism, while this study was hypothesis driven, based on functional evidence of a bone-body composition phenotype from an animal model. The stronger effects observed in the animal models, possibly reflect that a knock-out of the gene provides more extreme phenotypes and/or that mice over 1 year old are not a perfect model for aged humans. Secondly, not all phenotypes were available in both cohorts, i.e. fractures in young women were not included due to low prevalence. Thirdly, we cannot generalize if the results are applicable to other ages, ethnicities or men. In particular, *RAMP3* expression is potently regulated by estrogen, and contains functional estrogen receptor response elements in its promoter (Watanabe et al., 2006), hence further studies in women around menopause and at other time points thereafter, in other large cohorts would be of interest.

Strengths of the study include the comprehensive and extensive body composition and bone data collected in both cohorts, and the design of the cohorts - each containing approximately one thousand women with a homogeneous origin and residency, thus limiting potential bias from population stratification and environmental exposures. The single age of each cohort also minimizes confounding. This design, and the long duration of follow-up, allows investigation of temporal changes at different time windows in an effort to understand the contribution of genetic variation to bone health during the life-course. Furthermore, selection of RAMP3 variants is based on a biological rational and identification of a phenotype in an animal model assuming shared pathways, rather than from GWAS; and the SNP's studied, broadly encompass the gene region.

We conclude that polymorphisms in the *RAMP3* gene may contribute to the age-associated changes of body composition and risk of fracture, however needing further exploration of the underlying mechanisms.

The following is the supplementary data related to this article.

Supplementary Table S1 RAMP3 rs2074654 association with *BODY COMPOSITION CHANGE OVER TIME* in older women (OPRA cohort) Supplementary Table S1
