# Supplementary material for: Analysis of RAMP3 gene polymorphism with body composition and bone density in young and elderly women
Source: Gene X. 2019 Feb 14;2:100009. doi: 10.1016/j.gene.2019.100009 (PMC7286079; doi:10.1016/j.gene.2019.100009)
Supplement: Supplementary Table S1 — RAMP3 rs2074654 association with BODY COMPOSITION CHANGE OVER TIME in older women (OPRA cohort) [file mmc1.docx]

**Supplementary Table S1 RAMP3 rs2074654 association with *BODY COMPOSITION CHANGE OVER TIME* in older women (OPRA cohort)**

| **OPRA**  **5 year change (between 75y-80y)** | **rs2074654 ‘TT’ Homozygotes (n=585)** | **rs2074654 ’CC’ Allele Carriers**  **(n=47)** | **P value^a^** |
| --- | --- | --- | --- |
| Change in fat mass |  |  |  |
| Total Body (kg) | -0.65 ± 0.18 | -0.63 ± 0.49 | 0.775 |
| Trunk (g) | -124.9 ± 95.3 | -13.43 ± 25.3 | 0.523 |
| Leg (g) | -354.2 ± 64.5 | -334.87 ± 216.9 | 0.819 |
| Change in Lean mass |  |  |  |
| Total Body (kg) | 0.840 ± 0.263 | 1.111 ± 0.675 | 0.504 |
| Trunk (g) | -24.3 ± 48.3 | -7.98 ± 18.3 | 0.187 |
| Leg (g) | 440.0 ± 30.0 | 550.0 ± 120.0 | 0.348 |

*Association analysed using the dominant model (comparing the major allele homozygotes Vs. heterozygotes + minor allele homozygotes). Reported values are Mean (Std Error); ^a^P-values calculated by Kruskal-Wallis*
